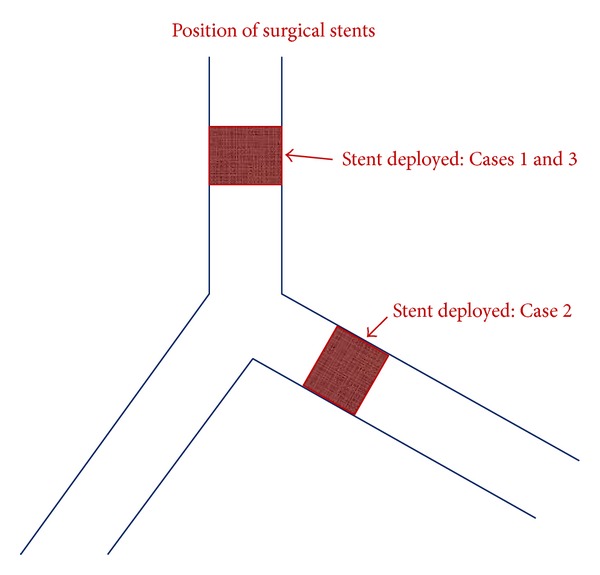# Anaesthesia for Tracheobronchial Stent Insertion Using an Laryngeal Mask Airway and High-Frequency Jet Ventilation

**DOI:** 10.1155/2013/950437

**Published:** 2013-08-18

**Authors:** Anne M. Dolan, Michael F. Moore

**Affiliations:** Department of Anaesthesia, Beaumont Hospital, Dublin 9, Ireland

## Abstract

An approach which promotes a rapid return to spontaneous respiration after tracheobronchial stent (TBS) insertion is considered the optimal one and is a belief shared by anaesthetists, respiratory physicians, and surgeons alike (Calvey and William (2008)). The value of the laryngeal mask airway (LMA), followed by use of the Monsoon 111 Acutronic jet ventilator pressure limiting system of ventilation, for the deployment of stents in the three individual cases that of tracheoesophageal fistula, a bronchoesophageal fistula, and tracheal compression from an invading oesophageal malignant tumour are reported. The roles of target controlled anaesthesia, high-frequency jet ventilation (HFJV), and the laryngeal mask airway in optimising the surgical field and reducing the risk of bronchospasm at emergence are advantages of this technique.

## 1. Introduction

This study comprised a retrospective analysis of how anaesthesia was performed for tracheabronchial stent (TBS) insertion in three unique cases of oesophageal and bronchial malignant pathologies. The institutional review board at our institution stated in the case of case studies ethics approval was not required. No components of our novel technique were regarded as experimental.

## 2. Materials and Methods

*Case 1*. A 56-year-old male patient, 71 kg in weight and height of 1.75 meters (BMI 25.3), presented with increasing dysphagia following a transhiatal oesophagectomy 9 months previously. Bronchoscopy revealed a tracheoesophageal fistula, and the decision to undergo TBS insertion was taken by his multidisciplinary team (upper gastrointestinal surgeons, ear, nose, and throat surgeons, clinical oncology services, and the palliative care team). 

Routine monitoring consisting of a pulse oximeter, noninvasive blood pressure monitor, electrocardiograph, airway gases: oxygen, carbon dioxide, vapour analyser, and airway pressure monitoring, in addition to intravenous access, were established. The patient was positioned in the semirecumbent position and preoxygenation commenced using an fractional inspired oxygen concentration (FIO_2_) of 1.0 for three minutes. Target controlled infusion (TCI) of propofol and a remifentanil infusion at a variable rate of 0.01–0.1 mcg/kg/min were commenced.

Propofol was titrated using the standard Marsh model [1]. Remifentanil was administered in a range of 0.01–0.1 mcg/kg/min at the same time. 

A laryngeal mask airway (LMA) classic (LMA North America, Inc. San Diego, CA, USA) size 4 was inserted, and the patient was gently hand-ventilated with oxygen and air while placed in the semirecumbent position. Positioning and ventilating via the LMA was done before muscle paralysis was administered to ensure that bag mask ventilation could be achieved with a supraglottic device, in the event of a difficulty with ventilation at a later stage during the surgical procedure, when stent deployment would be undertaken. The combined administration of oxygen and air was considered appropriate, while the LMA was in place, as it was felt that high oxygen concentrations were not indicated at this point in the procedure, given the knowledge that high levels of inspired oxygen concentrations for prolonged periods could be harmful (oxygen toxicity) on pulmonary function. Atracurium 0.5 mg/kg was then subsequently administered. After establishing muscle paralysis, the LMA was then removed, the surgical laryngoscope (Hopkins rod lens telescope—with attached video camera) was inserted by the ear, nose, and throat surgeons, and we the anaesthetists passed the jet catheter of the high frequency jet ventilator (Monsoon 111 Acutronic jet ventilator, Acutronic Medical systems AG, Fabrik im Schiffi, CH 8816 Hirzel, Switzerland) through the glottis beyond the tracheoesophageal fistula under direct vision. 

 The Monsoon 111 Acutronic jet ventilator was used for ventilation. The ventilator was set at a driving pressure of 1500 millimetres of mercury (mmhg), frequency of 150/cycles per minute, and an FiO_2_ of 1.0. Airway pressure was limited to 15 mmhg, and the peak inspiratory tract pressure was set at 11 mmhg.

**Figure 1:**
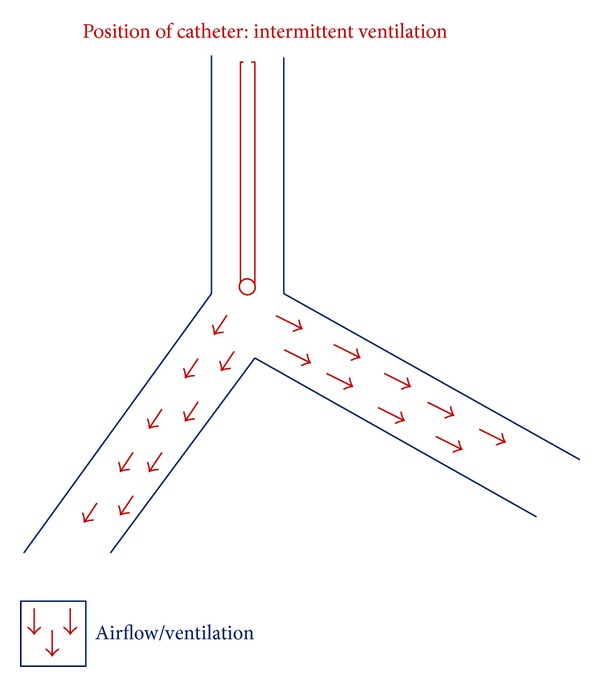


Jet ventilation was administered beyond the tracheaoesophageal fistula and in this way was successful in avoiding any contamination of the lung fields from gastric contents. At the time of stent deployment the jet catheter was withdrawn above the fistula and jetting ceased (see Figure 1). Apnoea was allowed to occur momentarily. Oxygen saturations were maintained above 90% and two boli of phenylephrine 100 micrograms (mcg) and 6 milligrams (mgs) of ephedrine were administered to maintain a target mean arterial pressure of 70 mmhg, during stent deployment. Mean arterial pressure dropped to a lowest of 60 mmhg during the course of the procedure. Once the stent was deployed across the fistula the jet catheter was then advanced through the stent under direct vision and jetting recommenced. Following confirmation of the correct position of the stent, the jet catheter was removed and the LMA was reinserted. Muscle relaxation was antagonised, by the administration of glycopyrrolate 2.5 milligrams (mgs) and neostigmine 500 micrograms (mcg). TCI was stopped and spontaneous breathing resumed via the LMA. The patient was then transferred to the recovery with the LMA in place, which on awakening was removed without incident, and subsequently the patient was transferred to the ward. The whole procedure took 65 minutes to be performed, including anaesthesia induction and emergence. Oxygen saturations were maintained between the 95 to 100 percent range. When saturations fell below this range the surgical procedure was interrupted and the fine bore catheter of the high-frequency jet ventilator system was inserted below the tracheoesophageal fistula to resume ventilation with the same settings, before the surgical procedure could be resumed.

*Case 2*. A 62-year-old man, 66 kg in weight and 1.5 m (BMI 30.0) in height presented with bronchial cancer, presented for stent insertion of his left main bronchus following diagnosis of a fistula between the left main bronchus and oesophagus.

**Figure 2:**
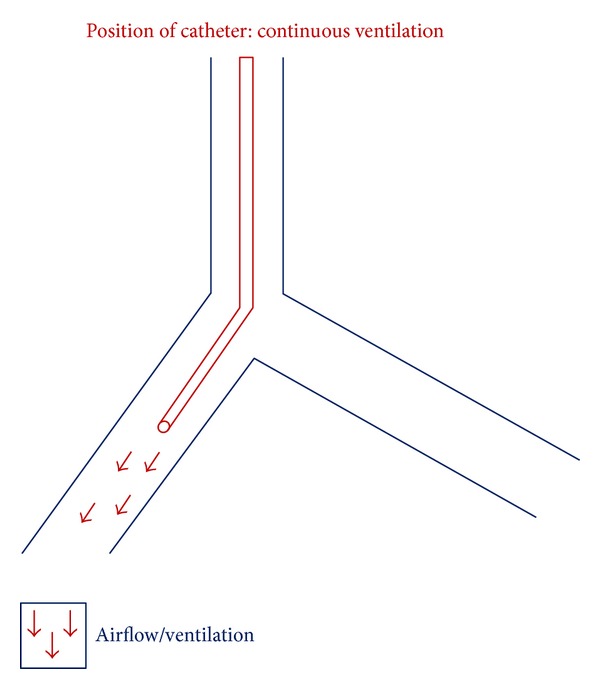


In the anaesthetic room, intravenous access was established, and with standard monitoring as reported in Case 1, the patient was preoxygenated for 3 minutes in the semirecumbent position. Anaesthesia was induced in the same way as in Case 1 until the LMA was inserted as before. Hand ventilation was again established using the LMA before administration of muscle relaxant as in Case 1 followed by removal of the LMA and insertion of the surgical laryngoscope. A jet catheter was then inserted under direct vision down the right main bronchus (Figure 2). Once in place 1 lung jet ventilation began using the Monsoon III Acutronic jet ventilator. Mean arterial pressure was maintained above 75 mmhg, and oxygen saturations were greater than 95% throughout this procedure with jet ventilation in progress. Ephedrine 9 mg was administered and 100 mcg of phenylephrine. MAP dropped to a lowest of 55 mmhg during the procedure, and the above medications were administered to maintain a MAP of greater than 70 mmhg. The surgical team deployed the stent across the bronchoesophageal fistula in the left main bronchus. Once the stent was deployed the jet catheter was withdrawn back through the right main bronchus into the trachea. The surgical laryngoscope and jet catheter were removed, and the LMA was reinserted followed by antagonism of the muscle relaxant with neostigmine 2.5 mg and glycopyrrolate 500 mcg. TCI was stopped and spontaneous ventilation resumed. The patient was placed in the semirecumbent position, self-ventilating through the LMA. The entire procedure took 45 minutes, and in this case there were no periods of apnoea, and ventilation was maintained throughout even during stent deployment as the fine jet catheter was passed down the opposing bronchus.

The patient was then moved to the recovery area, and the LMA was removed by the recovery nurse when he was fully awake. The patient returned to the ward without difficulty in intervening 30 minutes. There were no difficulties with ventilation afterwards.

*Case 3*. A 71-year-old lady, weight 66 kg, and height 1.6 m (BMI 25.7), presented with recurrence of an oesophageal tumour, resulting in tracheal stenosis and symptomatic stridor. She had a transhiatal oesphagectomy performed 2.5 years before. She presented for urgent tracheal stent insertion due to increasing respiratory distress and audible stridor during respiration at rest. 

As described in Cases 1 and 2, once intravenous access with standard monitoring was established, TCA was commenced and manual ventilation with the LMA size 4 was confirmed before muscle relaxant was administered. 

Subsequently the LMA was removed, the surgical laryngoscope as in Cases 1 and 2 was inserted by the ear, nose, and throat surgeons, and the jet catheter of the HFJV was placed under direct vision, into the trachea beyond the level of tracheal stenosis. Jet ventilation commenced. Prior to stent deployment the catheter was withdrawn above the compressed trachea under direct vision, similar to Case 1 (Figure 1). Jet ventilation ceased momentarily at the time of stent deployment to allow correct positioning of the stent but recommenced through the stent once it was inserted. The surgical technique in this case was complicated by a narrow tracheal lumen only 20% of its original patency. The surgical procedure was interrupted when oxygen saturations fell below 90% and the jet catheter was passed beyond the level of tracheal stenosis. 

 Mean arterial pressure (MAP) was maintained above 65 mmhg, and oxygen saturations, was maintained above 90% during stent deployment, with a heart rate maintained in the region of 70 to 90 beats per minute. Ephedrine 6 mg was administered for blood pressure that dropped to a MAP of 60 mmhg but otherwise was maintained above 65 mmhg. 

Intravenous dexamethasone 8 milligrams (mgs) was administered at induction to reduce further compromise in terms of airway oedema or the development of laryngospasm or indeed bronchospasm given the patient's clinical presentation of audible stridor on arrival to the anaesthetic induction room, signalling supraglottic airflow obstruction. 

The administration of glycopyrrolate 400 micrograms was also given at induction to reduce the patients oral secretions, in an attempt to improve the visual field down the airway and maximise conditions for stent deployment as well as jet ventilation.

The entire procedure took a total of seventy minutes to be performed, during which time the surgical procedure was interrupted and the fine jet catheter of the Monsoon ventilator system was inserted to resume ventilation and to maintain oxygen saturations above 90 percent range. 

Once stent deployment was satisfactory the surgical laryngoscope was removed and the LMA was reinserted as the patient was maintained in the semirecumbent position. Muscle relaxant was antagonised (by the administration of neostigmine 2.5 milligrams/glycopyrrolate 500 micrograms), and TCI were stopped. On return of spontaneous respiration the patient was transferred to the recovery room where the LMA was removed without difficulty. Postoperatively the patient continued to have stridor despite oxygen saturations in the 95% to 100% range on a 40% oxygen facemask. Adrenaline nebulisers were prescribed four hourly, and she was given twice daily doses of dexamethasone 8 mg. This continued for a further nine days, under the supervision of the ear, nose, and throat surgeons. The patient was monitored in a surgically monitored bed (where continuous pulse oximetry monitoring was available, and noninvasive blood pressure monitoring was performed). 

## 3. Discussion

The challenges to the anaesthetist of anaesthesia for tracheobronchial stent insertion are multiple and well documented, that of a shared airway, the patient with multiple comorbidities and advanced malignancy of the thorax, the possibility of failure because of the increasing pressure limitations within the already narrowed tracheal lumen available are just some of those reported [1, 2]. The low pressure ventilation offered by the Monsoon Cuatronic III jet ventilator controls the recoil pressure of the respiratory system which can be elevated at high lung volumes. 

The Monsoon is a time-cycled pressure limited ventilation system, and uses driving pressure rather than respiratory frequency to effectively eliminate carbon dioxide. The driving pressure is important, as it relates to mean alveolar pressure which in adults governs not only efficiency of oxygenation but also carbon dioxide removal. Being able to generate high frequency rates (greater than 80 cycles per minute) and the ability to monitor airway pressures in the respiratory tract are major advances in the safety of jet ventilator technology and could be considered as a highly effective means of carbon dioxide elimination.

While we recognise that even with an inspired concentration of oxygen (FIO_2_) of 1.0 once HFJV is initiated this same FIO_2_ does not mean that the partial pressure of oxygen in the trachea or proximal airway will be the same given the Venturi effect of air entrainment with the jet. We relied on oxygen saturations to determine how well ventilation was being performed. In retrospect our lack of invasive monitoring such as arterial blood gas monitoring which may have yielded greater benefit in appraising the effectiveness of HFJV in these three cases is a criticism our technique. We relied on normal oxygen saturation values and normal capnography values at induction when the LMA was used, to guide our management of the patient's ventilation. We also relied on the sophisticated Monsoon jet ventilator delivering time-cycled pressure limited breaths which operates using driving pressure rather than respiratory frequency to effectively eliminate carbon dioxide. 

 It is important to note that, in Case 1, jet ventilation did not commence until the jet catheter was positioned beyond the fistula in order to avoid gastric distension and potential soiling of the airway from gasrtic contents. Prior to stent deployment in Cases 1 and 3, jet ventilation was stopped momentarily, the catheter withdrawn above the fistula and level of stenosis to allow stent deployment while anaesthesia was maintained with TCA (Figure 1). Following stent insertion the catheter was reinserted through the stent and jet ventilation recommenced. 

The Monsoon Acutronic III jet ventilator was used for all three patients. It's fine jet catheter could be positioned through the surgical field under visual guidance, effectively ventilating without compromising the surgical procedure, even when positioned at distant sites within the trachea bronchial tree (Figure 3).

 Jet ventilation as a ventilator mode is believed to be due to the interaction of airflow under high pressure and involves a number of different velocity profiles [2]. 

Advantages of HFJV have been described and are threefold in terms of anaesthesia, surgery, and maintaining normal physiology. Reduced peak airway pressure, less haemodynamic compromise, reduced ADH and fluid retention, minimal vocal cord movement, greater visibility, surgical access, and versatility are some of those reported [2].

Ventilation could be easily performed under general anaesthesia and rapid emergence from anaesthesia attained. 

We observed that at the end of the procedure the insertion of the LMA facilitated the smooth return of spontaneous ventilation. There were no episodes of bronchospasm or coughing which could have resulted in displacement of the newly deployed stent. We believe the incidence of bronchospasm or coughing would be more likely if an endotracheal tube had been used after stent deployment. 

Similarly in Case 3 the presence of stridor would suggest inspiratory collapse of the posterior membranous tracheal wall, and ventilation seemed to be accomplished without difficulty with an LMA indicating that dynamic inspiratory obstruction played a significant role in this patient. However it was reasonable in this case compared to the previous two cases that dexamethasone was administered to reduce any further associated oedema of the posterior tracheal wall, given this clinical presentation. Likewise the administration of glycopyrrolate 200 micrograms to reduce oral secretions within the oropharyngeal tract was given at induction, to avoid impairment of the visual field which would be disadvantageous during the surgical process of stent deployment.

 By using an LMA at induction we safely ascertained that gentle manual ventilation could be achieved with this device before the administration of muscle relaxant. With this knowledge we were able to use the LMA again, at the time of transitioning from controlled to spontaneous ventilation, just before emergence from anaesthesia, and this was achieved without difficulty.

## 4. Conclusion

Our anaesthetic technique demonstrates adherence to airway safety. By establishing if an LMA could be used to ventilate the patient before muscle relaxant was given was important given the pathology we were presented with. 

The benefits in terms of surgery by using the HFJV system which involved a fine jet catheter were improvements in the visual field for surgeon. It also meant that the HFJV catheter could be reinserted during attempts at stent deployment to ensure that oxygenation was maintained. We believe that the anaesthesia time and surgical time were reduced compared to if an ET tube were used, and the use of the LMA counteracted any incidence of trauma occurring in the trachea before and after stent deployment which could have resulted in bronchospasm or expectoration of the newly deployed stent. There was no local anaesthesia applied to the vocal cords pre induction or at any stage during anaesthesia. There was no incidence of bronchospasm at any stage or in any of the three cases. We attribute this to the combined use of the LMA and of remifentanil and propofol infusions together with adaptation of the appropriate patient positioning resulting in the smooth induction and emergence from anaesthesia, which we observed.

 One of the limitations of this paper is in the number of patients presented and the ability to generalise conclusions drawn to a larger sample size. We do conclude however that we found this technique to be advantageous in this particular group of patients. Our use of the LMA has been generalised to a wider group of patients requiring tracheal stent insertion under general anaesthesia, over the last two years. We believe our technique resulted in benefits in terms of surgical outcome in addition to reducing recovery time, and there were no prolonged hospital stays or intensive care admissions, in patients with a terminal illness.

## Figures and Tables

**Figure 3:**